# Contextual Anonymization for Secondary Use of Big Data in Biomedical Research: Proposal for an Anonymization Matrix

**DOI:** 10.2196/medinform.7096

**Published:** 2018-11-22

**Authors:** John Rumbold, Barbara Pierscionek

**Affiliations:** 1 School of Science and Technology Nottingham Trent University Nottingham United Kingdom

**Keywords:** anonymization matrix, big data, data protection, privacy, research ethics

## Abstract

**Background:**

The current law on anonymization sets the same standard across all situations, which poses a problem for biomedical research.

**Objective:**

We propose a matrix for setting different standards, which is responsive to context and public expectations.

**Methods:**

The law and ethics applicable to anonymization were reviewed in a scoping study. Social science on public attitudes and research on technical methods of anonymization were applied to formulate a matrix.

**Results:**

The matrix adjusts anonymization standards according to the sensitivity of the data and the safety of the place, people, and projects involved.

**Conclusions:**

The matrix offers a tool with context-specific standards for anonymization in data research.

## Introduction

### The Promise of Big Data Research

The era of big data, which is rendered possible by high-power computing and increasingly cheap data storage, offers possibilities for research that have broad and lasting impact. In the last decade, the cost of memory has dropped from dollars per gigabyte to cents per gigabyte [1]. In 2013, the entire amount of data storage globally was 4.4 zettabytes (10^21^bytes), but in Utah, the National Security Agency facility’s storage capacity alone is now estimated at over 1 yottabyte (10^24^bytes). Traffic on the internet has now surpassed 1 zettabyte per year [2]. The use of data has the potential to transform many fields with health care as a leading prospect [3,4]. Vast amounts of health care data are already gathered, although not always in an electronic form. The widespread adoption of smartphone apps and wearables will vastly increase the amount of wellness and health data produced. Big data and databank research qualitatively differs from most other forms of health care research. Health data already collected for other purposes is often repurposed as a secondary use. This involves considerable cost savings but introduces the problem of lack of participant consent for research. Such issues are particularly acute with health care and other sensitive data. The potential is enormous, but the benefits are not fully exploited because of issues with consent, even though the research involves minimal risk to participants in most cases [5].

### Consent, Privacy, and Inconsistent Standards

Minimal risks, however, do not justify a cavalier approach to public consultation or consent requirements, as the failure of United Kingdom (UK) Care.data project demonstrated [6,7]. Failure to consult or inform the public properly resulted in the program being shelved despite having a firm statutory basis to proceed (although the relevant legislation may be incompatible with the General Data Protection Regulation (GDPR; see Multimedia Appendix 1) [8]). Several commentators have stated that the “consent or anonymize” model does not work for big data [5,9,10]. These issues have led to inconsistent decision making by governance bodies, which have the potential to hinder research in this rapidly progressing area. This paper justifies an anonymization matrix to guide decision making by research ethics review bodies. It draws on relevant norms in the European Union (EU) but will be applicable in other milieux.

Different standards for data research governance in different jurisdictions cause multinational projects certain issues, which have been addressed in the literature. There is also strong anecdotal evidence for inconsistency in approach among research governance bodies within the same jurisdiction. Reasons for such differences need exploration to ascertain whether the consistency and quality of decision making could be improved. Also pertinent is the consideration of public attitudes to inform decision making by research governance bodies.

## Methods

### Overview

A scoping study was performed using a recognized 5-step methodology to examine the regulation of data science in North America and the EU [11]. These jurisdictions were chosen because of their cultural connections and ease of access to literature because there were no resources to examine or translate publications in languages other than English. The major relevant statutes are GDPR (EU) and the Health Insurance Portability and Accountability Act (HIPAA; USA; Multimedia Appendix 1). GDPR provides derogations for conduct of research without consent, and these are much narrower in scope for health care and other sensitive data. The EU definition of anonymization is based on the possibility of reidentification using techniques that are “likely reasonably” to be used and without using of additional information (which may be easy to obtain). HIPAA provides a framework for anonymization that is far more prescriptive. The research question for the scoping study was as follows: what research on ethics of data anonymization exists to address public expectations of data management by researchers?

Studies were identified using an electronic search using Google Scholar, Westlaw, PubMed, and citation tracking and by manual search. Titles were selected after examination of abstracts. Data were charted from the small number of relevant studies selected with a narrative review generated from these papers. Results were collated and summarized and are presented in the analysis that forms this paper’s main text. This analysis and its subsequent conclusions have informed the construction of the proposed anonymization matrix.

### Health Care Data Research: What Are the Issues?

#### Consent

Health data research presents particular ethical issues. Large numbers of participants are exposed to minimal risks with a large number of projects possible using the same resource. Research tissue banks raise similar matters, and there is a considerable crossover with databank research because tissue banks usually have patient data in conjunction with tissue samples; for example, the UK Biobank project has approximately 500,000 participants and each participant, in addition to providing blood, saliva, and urine samples, completes an extensive lifestyle questionnaire [12]. Their imaging study aims to enroll 100,000 participants [13]. These resources are established for future research, the nature of which cannot be predicted [14,15]. The biobank is the curator of the resource rather than the body that performs research. A large number of participants and potential projects would make obtaining specific consent in each case a massive administrative burden and would inevitably reduce the amount of research performed within a specific cost envelope. Given participants’ altruism and minimal risks, if appropriate governance mechanisms were in place, that broad forms of consent are permissible is generally accepted [16-21]. These take several forms:

- Simple broad consent with the possibility of withdrawal at a later date: this suffers the disadvantage that the participant may not be kept aware of further projects to be able to exercise the right to withdraw consent
- Categorical consent: this is narrower—consenting to research in particular areas, which would be compliant with GDPR
- Consent to a form of governance: regulation of the resource can be entrusted to a reflexive governance mechanism that participants trust to make surrogate decisions informed by input of both shareholders and stakeholders [14,22]
- Combinations of these options or some other variation [23,24]

Alternatively, dynamic consent may be required. In this situation, participants are provided with information about each research project to decide whether to provide consent [25]. This mandates specific informed consent for each project, but it has been shown that participants can find this process too demanding [22,26-28].

When research is performed using “found data,” the issue of obtaining even broad consent is more problematic [29]. These considerations mean that with appropriate approval, participants’ informed consent may not be necessary, contrary to established practice in biomedical research [10,30]. There are broad research exemptions for data science, but derogations to permit research using sensitive data are narrower. GDPR states that research on sensitive data must be “in the public interest” (Recital 53).

There is the potential for several bodies to be involved with decision making on consent to use health care data for research—research ethics committees or institutional review boards, data access committees, data protection authorities, and health service management boards. Some of these bodies have local, national, and supranational arms, each of which may have a different perspective and make different decisions based on the same facts. There are anecdotal reports of divergent opinions on consent to data use between research ethics committees and the Confidentiality Advisory Group of the UK’s Health Research Authority (Personal communication from John Fistein). Although the Confidentiality Advisory Group’s main remit is to advise the Health Research Authority and Secretary of Health pursuant to the Health Service (Control of Patient Information) Regulations 2002 and s251 of the Health and Social Care Act 2012, its assessments include an implicit ethical evaluation of whether confidential patient information can be processed. Similar inconsistencies and tendencies toward risk aversion have been described in relation to administrative data [5].

Potential harms that participants in data research might be exposed were examined in a scoping study conducted at the Mason Institute and Farr CIPHER for the Nuffield Council on Bioethics Working Party on Biological and Health Data and by the Wellcome Trust’s Expert Advisory Group on Data Access [31]. Limited owing to time and resource constraints, their study focused on the instances of financial damage and emotional distress to individuals. There may be substantial harm to organizations as well, including reputational damage and loss of trust. Many incidents they identified were related to maladministration, and this reinforces the need for secure systems for data science.

Difficulties with consent illustrate that merely gaining consent is not a panacea for all data research issues even when practicable. The standard paradigm for data research is to “consent or anonymize.” Therefore, if consent is not practicable for big data projects, the researcher might choose to anonymize data. This is not necessarily straightforward and introduces a host of other issues.

### Anonymization

Anonymization is a means of preventing a breach of confidentiality and preserving privacy. Anonymized data are not protected under data protection law. Confidentiality and privacy are related concepts: confidentiality is a *duty* owed, often by a professional, to an individual in particular circumstances; privacy is a *right* that a person enjoys. An individual divulges many sensitive facts to professionals, particularly in law and medicine [32], with the understanding that the professional has a professional, legal, and ethical duty to maintain the information and data in confidence or face hefty sanctions for breaching these duties [33-35]. Duty of confidentiality does not apply where data have been anonymized. A duty of confidentiality is included in the Hippocratic Oath [36] and the Geneva Declaration [37], but there is an additional duty in the Geneva Declaration which is to: “share my medical knowledge for the benefit of the patient and the advancement of healthcare.”

This injunction could be interpreted as placing a duty on physicians to share data for purposes of medical research when conducted for the common good. In the UK, the Information Commissioner’s Office (ICO) and the Caldicott Review have commented on the problem of not using data [38,39]. Caldicott made this an added seventh principle: “The duty to share information can be as important as the duty to protect patient confidentiality.” Although this added principle is in the context of particular duties to an individual, rather than research *per se*, it could be interpreted to include a duty to use data to improve health care.

The distinction between privacy and confidentiality is acknowledged in data protection law in which particular protections apply to those “who in the circumstances owe a duty of confidentiality which is equivalent to that which would arise if that person were a health professional” (UK Data Protection Act 1998 (Multimedia Appendix 1); similar provisions apply in other transpositions of the Data Protection Directive). Data safe havens require researchers’ contractual duty to maintain confidentiality and not to attempt reidentification [40,41]. Hefty sanctions should be applied only to those intentionally breaching guidelines; otherwise, a tendency will arise to restrict data sharing unnecessarily [31]. This is one factor behind the tendency of not sharing data when doing so is both legally and ethically acceptable [5].

Anonymization is the procedure that removes data from the remit of data protection law, which pertains only to personal data. Data about or relating to a person are no longer personal if these cannot be linked to the person. Anonymization requires more than just removal of identifiers; the combination of 3 pieces of data could identify 87% of US residents—5-digit zip code, birth date, and sex (note that this would not satisfy the HIPAA Privacy Rules anonymization criteria for 2 of the 3 fields, see Multimedia Appendix 2) [42].

GDPR defines personal data as relating to “an identified or identifiable natural person (‘data subject’),” as included in Article 4.1. The definition of “an identifiable natural person” covers identification by direct or indirect means and can range from a name to social identity. The nature of personal data is not further defined. Although some facts about an individual are trivial, nonetheless, defining content of personal data that would cover all individuals in all situations and be universally acceptable is difficult. The UK Anonymisation Network (UKAN), run by a consortium of the University of Manchester, University of Southampton, the Open Data Institute, and the Office for National Statistics to establish best practice for anonymization, has classified data, as shown in Table 1.

Defining personal data purely by content is problematic, perhaps because some data tangentially refers to a person, for example, a vehicle registration plate (which would be secondary personal data under the UKAN schema), or because whether the data identifies someone depends on many other factors. This issue is illustrated vividly by the decision in *Breyer v Germany* on whether a dynamic internet provider address is personal data (Multimedia Appendix 1). UKAN states that anonymization depends not only on data but also on the environment within which that data are found [43].

**Table 1:** Four types of data depending on whether they are about people and whether they are identifiable [43]. Source: Anonymisation Decision-Making Framework.

About individuals	Nonidentifiable data	Identifiable data
Yes	Anonymized data	Primary personal data
No	Apersonal data	Secondary personal data

UKAN has divided anonymization into the following 4 types: formal, guaranteed, statistical, and functional [43]. First, formal anonymization means that direct identifiers have been removed. This does not satisfy the EU legal standard. Second, guaranteed or absolute anonymization, as the name suggests, provides security against any possible reidentification but often at the expense of the removal of large amounts of data. Data protection law does not require this but individual data controllers may deem it necessary. Third, statistical anonymization is tied to the concept of statistical disclosure control. It seeks to reduce the chance of reidentification to below a given predetermined statistical threshold. This threshold is crucial to whether anonymization provides real protection; for example, with differential privacy, the epsilon value selected by Apple has been severely criticized for providing little protection of privacy [44]. Finally, functional anonymization examines the risk of anonymization within a particular context, taking into account motivations of an attacker, consequences of disclosure, and data divergence among other criteria. Data protection legislation does not consider these factors in legal standards for anonymization.

### Difficulties With Anonymization

In light of difficulties in defining and ensuring anonymity, definitions of personal data across the globe are becoming broader [45-48]. Proliferation of data collected by various data controllers about data subjects and evolution of techniques to reidentify data subjects has required reassessment of anonymization. Now, unless data are substantially masked, swapped, grouped, or deleted, an individual can often be identified by someone sufficiently determined, with the right technical skills, and with sufficient additional data [42,49]. Although methods have been developed to achieve tailored levels of processing to maximize data’s utility, while ensuring anonymization to a given level, none of these alter the fact that making good use of data and achieving rigorous anonymization are currently incompatible. Statutes do not mandate guaranteed anonymization. Instead, they set a standard of difficulty of reidentification that must be achieved. This depends on factors such as motivations and skills of an intruder and information that might be combined with data. None of the legal standards appear to vary according to data’s circumstances or sensitivity, although these factors feed into an assessment of good practice [50,51] and could be incorporated into codes of conduct that would be part of the regulatory milieu encouraged by GDPR Article 40 (Comments made at the Privacy Engineering Research and the GDPR Workshop, KU Leuven November 10, 2017 as observed by JR).

Sensitive personal data are defined in data protection legislation, and health care data are one of those categories (s2, Data Protection Act 1998, UK). There are additional ethical and legal protections for health care data, which may include specific protections for particular categories, for example, sexually transmitted infections (eg, National Health Service, Venereal Diseases, Regulations SI 1974/29-UK, now repealed) and genetic data (eg, SI #687/2007—Data Protection Processing of Genetic Data Regulations 2007, Ireland; outside the EU, there is the Federal Act on Human Genetic Analysis 2004, Switzerland). It has been demonstrated that public conception of sensitive data categories may vary from that defined in legislation [52,53].

Anonymization introduces several problems for data researchers, particularly in health care research. It reduces the quantity and quality of usable data to a variable degree. Anonymization makes it impossible to verify data or act on any results that might have consequences for participants, for example, when imaging studies are performed. It will prevent linking of records, either to form a longitudinal health record or to link datasets on relevant issues such as socioeconomic indicators. Pseudonymization makes several of these objectives possible; however, in GDPR, pseudonymization is specifically excluded from being categorized as anonymization.

### Public Attitudes Toward Data Science

The basis of an ethical waiver for consent largely rests on the presumption that the public would consent to having their data used in this way, given the potential for creating public good. This necessitates an assessment of public attitudes. Different projects and datasets may require different approaches for preserving participants’ privacy, while maximizing the benefit of research performed. Another consideration is the public’s attitude toward data research, in particular, factors that affect the public’s expectation of how their data will be processed. This is especially important because the social license on which data research with consent or anonymization relies rests on public support.

The public’s attitudes toward use of data for research have been studied by the Scottish Health Informatics Programme (SHIP) and the Ipsos MORI Social Research Institute on behalf of several UK organizations [23,54-58]. Use of deliberative engagement methods has proven crucial because public attitudes to data sharing are affected significantly by provision of sufficient information on how data are used. During their deliberative engagement exercise, SHIP found that initially, members of the public expected consent to be asked for each research project. However, with greater understanding of the number of potential research projects with similarity of issues, they considered broad consent to be as acceptable, if not preferable. A similar result was found in a study of the US public [26]. In recent years, the Ipsos MORI Social Research Institute has conducted studies on behalf of the Medical Research Council (the use of personal health information in medical research, 2007) [23]; the Economic and Social Research Council (Dialogue on Data) [58]; the Wellcome Trust (Commercial access to health data, 2016) [56]; the Royal Statistical Society (Public attitudes to the use and sharing of their data, 2014 [55]); and the Government Data Science Partnership (Public dialogue on the ethics of data science, 2016) [57]. Similar to SHIP, it found that attitudes to data sharing varied considerably depending on the purposes and likelihood of public benefit.

Nissenbaum coined the term “contextual integrity” to denote the binding of data sharing practices to particular contexts [59]: The mere fact that data are in the public domain does not constitute license to disseminate them more widely. Solove also dealt with this issue in his taxonomy of privacy [60]. As the example of *Nader v General Motors Corp* demonstrates, intrusive monitoring of activities performed in public can be an invasion of privacy; just because Nader was in a bank did not permit anyone to know how much money he was withdrawing (nor, indeed, that he was there to withdraw money at all; Multimedia Appendix 1). Therefore, posting material on social media does not automatically make their use for research ethical. Anonymization may still be necessary and appropriate for Facebook and Twitter posts because posters had not intended their material to be disseminated to a wider audience.

With research on attitudes toward sharing location data in particular, Sadeh has also found that privacy is highly contextual [62-64]. Willingness to share location data depends on several factors including time of day, day of the week, social group requesting data, and location. Sadeh found that the purpose for which data would be used was particularly important in decision making. If location data are crucial to the central purpose, its use is much more frequently acceptable than when it is tangential or unrelated to the app’s central purpose. Similarly, an individual who may be willing to share sensitive data, such as in health care, might be unwilling to have socioeconomic data linked with those medical records [65]. This points to a demand for improved, granular consent requirements to reflect the need for data from individuals.

## Results

This proposal arises from an ethico-legal analysis completed during our work on the Aegle project. It takes into consideration recent EU legislation, but the resulting matrix is applicable to most jurisdictions.

## Discussion

### A Framework for Information Governance: A Proposed Solution

Governance is an inclusive form of regulation that encompasses governmental laws and regulations. Information governance frameworks require synthesis of data protection laws, guidance from national data protection officers, and an appreciation of expectations of the public they serve. Governance mechanisms can and ought to be more flexible and responsive than governmental laws and regulations. The main justification for the proposed matrix is all the evidence that supports the common sense notion that people are willing to share different amounts and types of data with different people, in different settings, for different purposes, at different times. Therefore, it is reasonable to conclude that using the same anonymization standard for all data protection and freedom of information purposes does not reflect societal attitudes to data or provide a governance framework that satisfies individuals’ reasonable expectations. A proportionate form of governance is preferable to protect individuals as fully as practicable while maintaining the capacity to generate useful insights from data [66,67]. The demands of good governance are usually greater than the legal standard, and this is particularly true for research in which standards are left for the scientific community to decide (eg, GDPR allows consent “in keeping with recognized ethical standards for scientific research,” Recital 33) [8]. However, there is no suggestion that this assessment can be done without public engagement.

The trust placed in medical practitioners and academic researchers therefore entails the public’s possible acceptance of a lower standard of anonymization, given that data users have a professional or contractual duty to respect confidentiality [35,36]. There is a persuasive case for having different standards of anonymization for medical research conducted in safe havens or at least by researchers under a duty (whether professional or contractual) of confidentiality, including a duty of not attempting reidentification, and for data released to the public whether under a freedom of information request or not. The UK Data Protection Act allows processing of medical data under Schedule 3, Para 8.1b by “a person who in the circumstances owes a duty of confidentiality which is equivalent to that which would arise if that person were a health professional.” The trustworthiness and motives of those who examine data are highly relevant to which precautions would be prudent. There is no control over what techniques can be used and by whom once data are released to the public and are therefore “in the wild.”

Data protection authorities have commented on the dynamic nature of personal data. The UK ICO (2012) noted that predictions about data available now or later cannot be made with certainty (page18) [39]. The EU’s Article 29 Working Party reached a similar conclusion, recognizing that the changing nature of data and its usage as well as growth in information that could aid identification can give rise to new data protection issues. This requires an appreciation that anonymization is not a one-off exercise [68]. Data that at one point in time is anonymized may subsequently be identifiable and thus become personal data once more. Based on these considerations and the fact that once data has been released to the public, we conclude that it cannot be recalled and operations performed cannot be limited in any way; there is justification for applying the most stringent standards of anonymization to data for public release.

This distinction was not considered by the UK ICO in their decision FS50565190 against Queen Mary University of London, relating to a trial of treatment for chronic fatigue syndrome. Their decision held that the University could not withhold data anonymized to the Information Commissioner’s satisfaction, despite concerns about activists trying to reidentify participants. The ICO wanted a specific explanation as to how reidentification would be achieved [69]. Section 22A of the Freedom of Information Act now exempts research studies from disclosure prior to publication of papers, but this only extends the timeframe for disclosure rather than absolutely exempting them. The University argued that participants had an expectation that their data would be confidential and that, in a small community, addition of information, for example, about hospital appointments, might enable reidentification. Participants had already withdrawn consent because of such fears, and this required expensive, time-consuming reanalysis of the remaining data.

In summary, we argue that the evidence demonstrates that neither consent nor anonymization to current legal standards is a solution to all data research issues. Limitations of anonymization make the application of the same standard across the board problematic. Recognition of the current framework’s inadequacy has led us to propose an anonymization matrix for treatment of sensitive data, particularly health care data. Our hypothesis is that the matrix will improve proportionate information governance and can therefore improve the trustworthiness and utility of data research. This hypothesis requires testing with empirical research, which is beyond the remit of this paper.

### Proposal

#### An Anonymization Matrix

A tool for research ethics committees, institutional review boards, and data access committees for assessing data protection aspects of a project and achieve consistent proportionate information governance is proposed. This P-R matrix (Table 2) includes a number of levels of anonymization adjusted according to the best evidence about public attitudes to trustworthiness, particularly recent research on public attitudes about data’s use for research. The matrix also takes into account the unpredictability of health care data’s future identifiability, holding that any data for public release should be subject to the highest standards of anonymization in accordance with the precautionary principle. GDPR and ethical standards demand that when research is not in the public interest, the standard paradigm of “consent or anonymize” should apply.

Levels 1-3 of anonymization referenced in the table are defined in Multimedia Appendix 3. They are developments of the HIPAA Privacy Rule (detailed in Multimedia Appendix 2) with the addition of algorithm-based methods that can adjust processing according to required levels of k-anonymity, l-diversity, t-closeness, and differential privacy. Definitions of particular categories and rationales are provided below. We have also incorporated a UK adaptation for the obscuration of postcodes in Multimedia Appendix 4.

Considering that particular contexts may make an individual more vulnerable or the attempts to breach more skilled or more determined is also appropriate. These special circumstances include data on celebrities or other persons about whom a considerable amount of information is already in the public domain and is widely known. Use of metrics to determine the possibility of reidentification is appropriate, although the Level 3 standard combined with anonymization algorithms to provide k-anonymization may not be sufficient to allow for public release. Synthetic data, or a dataset that has been subtly altered from the original, is a good substitute in many situations because it can be demonstrated to provide results very similar to those obtained from data on which it is based [70,71]. The content of the columns and rows in Table 2 is explained further.

**Table 2:** P-R anonymization matrix.

Context of data	Data use authorized without consent^a^	Health care data use without consent	Very sensitive health care data^b^ use without consent	Special circumstances without consent
Research in safe havens^c^	Anonymization not required	Level 1	Level 1	Level 2
Research to which duty of confidentiality applies	Anonymization not required	Level 1	Level 2	Level 3
Research to which no duty of confidentiality applies^d^	Level 1 + algorithmic manipulation^e^	Level 1 + algorithmic manipulation	Level 2 + algorithmic manipulation	Level 3
Information for public release^f^	Level 3 or synthetic data or no release	Level 3 or synthetic data	Level 3 or synthetic data	Level 3 or synthetic data or no release

^a^Where authorization for data processing without consent has been provided by a specific statutory body, a body that provides appropriate safeguards, or the equivalent for research ethics. These bodies have powers to authorize data use without anonymization; however, good practice requires data minimization with justification for inclusion of all identifying data.

^b^Very sensitive data are not exhaustively defined in this paper because they depend heavily on particular sociocultural sensitivities; for example, alcoholic liver disease would be a sensitive diagnosis in some cultures but not necessarily in all. Sexually transmitted infections are usually considered very sensitive. Public consultation is needed on use of health care data in an ongoing process.

^c^Requirements for accreditation include that researchers are under contractual duties of confidentiality, including not to attempt reidentification [40].

^d^It should be noted that the UK government has signaled an intention to create a new criminal offense of reidentification [72]; other jurisdictions, including New Zealand, Australia, and Canada, are also considering this [73,74]. Currently, reidentification would be merely a breach of data protection law.

^e^Algorithmic manipulation means data masking, clustering, or deletion to satisfy demands of k-anonymity and other metrics such as l-diversity, t-closeness, or differential privacy.

^f^As noted above, the UK Information Commissioner’s Office could compel release under the Freedom of Information Act 2000 of data only anonymized to their standard (currently, the motivated intruder). This standard is arguably deficient for public release of health data [61], and we propose statutory change to enable an appropriate level of privacy protection to be required.

#### Rationale for the Anonymization Matrix

Authorization means that data use has been permitted without consent by a statutory body, research ethics committee, or other empowered governance body. “Duty of confidentiality” in this instance means a professional or contractual duty of confidentiality equivalent to those of health care professionals, additional to a duty of not attempting reidentification.

### Research in Safe Havens

Several requirements must be met for a data safe haven to be accredited.

1) Authorization by the appropriate body covers the use of data for research without any anonymization, whether in a data safe haven, when the researcher is bound by a duty of confidentiality in the same way as medical professionals.

2) and 3) Anonymization is required to meet legal requirements where authorization has not been granted. Because research is being conducted in safe havens, there is no requirement for a standard higher than Level 1.

4) Where there are special circumstances, it seems reasonable to expect a higher standard of anonymization because reidentification could occur spontaneously without any deliberate attempt by the researcher.

### Research Where Duty of Confidentiality Applies

Duty of confidentiality provides protections for participants but not other safeguards provided in accredited data safe havens. Hence, some additional anonymization may be necessary.

1. Where authorization is granted, there is no need for anonymization.
2. Where no authorization has been granted, Level 1 anonymization will satisfy legal and ethical requirements.
3. Where data are particularly sensitive, risks related to disclosure are correspondingly higher. Therefore, we recommend Level 2 anonymization.
4. Where there are special circumstances, Level 3 anonymization reduces risk of inadvertent reidentification.

#### Research in Which No Duty of Confidentiality Applies

If researchers are not under a duty of confidentiality, safeguards to prevent reidentification should be stronger. Excessive processing of data can be reduced by resorting to algorithmic manipulation.

1. Although there is no legal requirement when authorization has been granted for any anonymization to be performed, we argue that where there is no duty of confidentiality, ethical bodies should require it. Additionally, algorithmic manipulation should be required to ensure that reidentification cannot occur.
2. Here anonymization is legally required. The safeguard of additional algorithmic manipulation should be required by ethical bodies.
3. Processing of more sensitive health care data warrants the higher level of anonymization with algorithmic manipulation.
4. Research on data in special circumstances where researchers are not bound by a duty of confidentiality is worthy of the highest levels of anonymization (where such research is permitted at all).

#### Public Release

When information is released to the public, anonymization must be as rigorous as possible, owing to future development of new techniques for reidentification and possible release of further information. For this reason, we recommend that at least Level 3 anonymization be used. Synthetic data are preferable when substitution is feasible.

1) and 4) Without consent or with special circumstances, there is a case for not releasing any data to the public. Synthetic data pose no privacy risk.

2) and 3) Release of rigorously anonymized data are acceptable although synthetic data are preferable.

These requirements would need periodic review. Because data for public release cannot be modified to increase protection from reidentification, standards for anonymization must be robust enough to provide protection for at least the medium term. The proposed matrix provides guidance for research ethics review bodies to harmonize their ethical assessments with data protection requirements, while providing the enhanced protection expected for sensitive data.

### Techniques of Anonymization

It has been said many times that “ought” implies “can” [75]. Anonymization is not an all or nothing process, but rather a spectrum of processing that provides greater or lesser degrees of difficulty in reidentifying an individual. Although finite risk is associated with nearly all data science research, the public is willing to accept this if mitigated by appropriate data security and safeguards [76,55]. A further solution to the problem of reidentification is to restrict access to researchers who have given assurances that they will not attempt reidentification. Although potentially attractive and reassuring to the public, this currently makes no difference to whether data are classified as indirectly identifiable. However, such assurances would be good evidence of the provision of appropriate safeguards by the body concerned.

Techniques involved in anonymization reduce utility of data to a greater or lesser extent [51,77,78]. Academic literature has much debated risks associated with anonymized data. Although researchers have demonstrated that datasets can be reidentified in defined circumstances, whether these scenarios reflect what is likely in the real world is contentious [42,49,79].

The 2 approaches to anonymization are rule based and risk based. Rule-based anonymization is typified by the first part of the HIPAA Privacy Rule, which mandates obscuring particular data fields. The HIPAA Privacy Rule is easy to apply, but there are problems with it. In some circumstances, it involves unnecessary deletion of data; in others, it fails to provide adequate protection from reidentification [80]. Ruling out unusual data that can uniquely identify an individual is difficult, an example being the mayor of Ottawa [81]. There is also the issue of where sufficient data are available in the public domain about an individual for reidentification to be feasible [61].

Risk-based anonymization involves processing guided by the calculation of the risk of disclosure and assessing which data need to be obscured to achieve this [50]. It would include the statistical expertise-based standard in the HIPAA Privacy Rule and involves such techniques as k-anonymization, l-diversity, t-closeness, and differential privacy [82-85]. These techniques examine data for patterns that would enable reidentification (if, eg, one dataset has particular attributes) and selective data masking, clustering, or deletion to reduce the possibility of drawing inferences from deidentified data. These techniques reduce deterioration in data, but they do not eliminate it. If too large a value for epsilon is selected for differential privacy, then privacy protection will be minimal [44]. An overstringent rule-based approach to anonymization is problematic, and a proportionate form of governance has distinct advantages [15,86]. If researchers agree to not attempt reidentifying participants and their duty is reinforced by the prospect of sanctions, it provides reassurance and facilitates preservation of intact data. Conversely, data for public release may be subject to any number of techniques and addition of data from a variety of sources, both legal and illegal [61,87].

Advances that have enabled reidentification also enable other inferences from existing data. This is, after all, the basis for linkage research. One of the most famous examples is the Target customer being sent offers on baby-related items when she had not yet told her father of her pregnancy. The supermarket had inferred the fact of her pregnancy from her purchasing habits [88]. The participant cannot have given permission for the production or storage of these new facts when consent has not been specifically given for research purposes. Recently, ICO fined charities for conducting “wealth screening” of potential donors [89].

### Conclusions

The literature on privacy and attitudes toward use of data for research purposes provides support for application of different standards of anonymization depending on circumstances. Additionally, the regulatory burden can be reduced by harmonization of criteria applied by research ethics committees and other governance bodies. For research ethics and data access committees, our anonymization matrix provides guidance that exceeds the requirements of current data protection laws. Each row and column of the matrix corresponds to a meaningful ethico-legal distinction. It offers contextual guidance for research ethics bodies to recommend appropriate levels of anonymization when gaining specific consent is not feasible.

We propose that research ethics bodies should not deny permission on grounds of privacy or consent issues for projects that satisfy these anonymization requirements. Satisfying these requirements should make approval, for example, by the Confidentiality Advisory Group easier. Additionally, compliance with standards that exceed legal requirements help secure the social license and thus ensure data bank projects’ legitimacy and longevity.

The major potential advantage of such a matrix is the facilitation of international projects. Any ethico-legal framework that satisfies the requirements of multiple jurisdictions without imposing excessive regulatory burden will be a valuable tool for such projects. To demonstrate the matrix’s value for improving research ethics committees’ decision making on information governance, we propose its use in EU data science projects on a trial basis.

## Appendix

Multimedia Appendix 1 Statutes and cases.

Multimedia Appendix 2 The HIPAA (Health Insurance Portability and Accountability Act) privacy rule.

Multimedia Appendix 3 Anonymization standards proposed levels 1-3.

Multimedia Appendix 4 UK modification of geographic rule.

## Abbreviations

GDPR: General Data Protection Regulation
HIPAA: Health Insurance Portability and Accountability Act
ICO: Information Commissioner’s Office
SHIP: Scottish Health Informatics Programme
UKAN: UK Anonymisation Network
EU: European Union
