# Supplementary material for: Contextual Anonymization for Secondary Use of Big Data in Biomedical Research: Proposal for an Anonymization Matrix
Source: JMIR Med Inform. 2018 Nov 22;6(4):e47. doi: 10.2196/medinform.7096 (PMC6284146; doi:10.2196/medinform.7096)
Supplement: Multimedia Appendix 1 [file medinform_v6i4e47_app1.pdf]

Multimedia Appendix: Statutes and Cases

Statutes

Data Protection Act 1998 c 29

General Data Protection Regulation (EU) 2016/679

Health Insurance Portability and Accountability Act 1996 Pub.L. 104-191

Cases

Breyer v Germany Case C-582/14 ECLI:EU:C:2016:779

Nader v General Motors Corp., 25 N.Y.2d 560, 255 N.E.2d 765, 307 N.Y.S.2d 647 (N.Y. 1970)
