# Supplementary material for: Contextual Anonymization for Secondary Use of Big Data in Biomedical Research: Proposal for an Anonymization Matrix
Source: JMIR Med Inform. 2018 Nov 22;6(4):e47. doi: 10.2196/medinform.7096 (PMC6284146; doi:10.2196/medinform.7096)
Supplement: Multimedia Appendix 2 [file medinform_v6i4e47_app2.pdf]

Multimedia Appendix 1

The HIPAA Privacy Rule

“A covered entity may determine that health information is not individually identifiable health information only if … the following identifiers of the individual or of relatives, employers, or household members of the individual, are removed:” (HIPAA, 1996)

A) Names;
B) All geographic subdivisions smaller than a State, including street address, city, county, precinct, zip code, and their equivalent geocodes, except for the initial three digits of a zip code if, according to the current publically available data from the Bureau of the Census: (1) The geographic unit formed by combining all zip codes with the same three initial digits contains more than 20,000 people; and (2) the initial three digits of a zip code for all such geographic units containing 20,000 of fewer people is changed to 000;
C) All elements of dates (except year) for dates directly related to an individual, including birth date, admission date, discharge date, date of death; and all elements of dates (indicative of such age, except that such ages and elements and elements may be aggregated into a single category of age 90 and older;
D) Telephone numbers;
E) Fax numbers;
F) Electronic mail addresses;
G) Social security numbers;
H) Medical records numbers;
I) Health plan beneficiary numbers;
J) Account numbers
K) Certificate/license numbers;
L) Vehicle identifiers and serial numbers, including license plate numbers;
M) Device identifiers and serial numbers;
N) Web Universal Resource Locators (URLs);
O) Internet Protocol (IP) address numbers;
P) Biometric identifiers, including finger and voice prints;
Q) Full face photographic images and any comparable images; and

R) Any other unique identifying number, characteristic, or code

Also that

ii) The covered entity does not have actual knowledge that the information could be used alone or in combination with other information to identify an individual who is a subject of the information.

Alternatively, there is a statistical standard where:

> A person with appropriate knowledge of and experience with generally accepted statistical and scientific principles and methods for rendering information not individually identifiable, [a]pplying such principles and methods, determines that the risk is *very small* that the information could be used, alone or in combination with other reasonably available information, by an anticipated recipient to identify an individual who is a subject of the information; and [that person] documents the methods and results of the analysis that justify such determination. [italics authors']

This latter standard is rarely used because of uncertainty over which persons have appropriate knowledge and experience.
