# Supplementary material for: Contextual Anonymization for Secondary Use of Big Data in Biomedical Research: Proposal for an Anonymization Matrix
Source: JMIR Med Inform. 2018 Nov 22;6(4):e47. doi: 10.2196/medinform.7096 (PMC6284146; doi:10.2196/medinform.7096)
Supplement: Multimedia Appendix 3 [file medinform_v6i4e47_app3.pdf]

Multimedia Appendix 2

Anonymisation standards proposed **Levels 1-3**

**HIPAA Category**	**Level 1 additions**	**Level 2 additions**	**Level 3 additions**
**B**	Local modifications according to population density and coding etc (see Appendix C for UK example)		Age groups, rather than ages in years
**F**	Also social media accounts and/or usernames, Skype handle, etc		
**P**	Pictures not included unless processed for biometric purposes		
**Q**	Eye masking insufficient to anonymise photographs(International Committee of Biomedical Photographers, 2005)	Photographs anonymised even to those who know the individual only allowed. This applies to facial photos plus others eg where a distinctive tattoo is included	
**R**		Sufficiently rare characteristics by themselves can be indirect identifiers. This includes rare diagnoses which should be grouped by category.	
**[S]** *information about occupation*			No occupations except by broad categories
**ii)**			Neither actual knowledge nor *strong grounds to suspect* an individual could be identified
