# Supplementary material for: Contextual Anonymization for Secondary Use of Big Data in Biomedical Research: Proposal for an Anonymization Matrix
Source: JMIR Med Inform. 2018 Nov 22;6(4):e47. doi: 10.2196/medinform.7096 (PMC6284146; doi:10.2196/medinform.7096)
Supplement: Multimedia Appendix 4 [file medinform_v6i4e47_app4.pdf]

Multimedia Appendix 3

UK modification **of** geographic rule

List postcode districts (first part [outward code] including letter(s) and 1 or 2 numbers) except where this district contains less than 20,000 people according to 2011 census then just include the letter(s).

Equivalent modifications can be adopted for other European countries to ensure no geographical regions with a population of under 20,000 are identified. Where there is low population density and many postcode districts are under 20,000 people, alternative codes can be devised to be more specific about area. Alternatively, other metrics can be substituted e.g. Index of Multiple Deprivation or Jarman Score. The EU collects statistics on income and living conditions (EU-SILC), which will be the most useful source for indices of socio-economic deprivation as it covers all the countries concerned.
